# Virtual reality-based training to augment recovery of hand dexterity after surgery for degenerative cervical myelopathy

**DOI:** 10.1038/s41598-025-05793-5

**Published:** 2025-07-02

**Authors:** Viprav B. Raju, Roxanne Hauer, Mohammad Ghassemi, Anjishnu Banerjee, Derek Kamper, Brian D. Schmit, Aditya Vedantam

**Affiliations:** 1 Department of Neurosurgery, Medical College of Wisconsin, 8701 Watertown Plank Road, Milwaukee, WI 53226 USA; 2 Joint Department of Biomedical Engineering, Marquette University- Medical College of Wisconsin, Milwaukee, WI USA; 3 Joint Department of Biomedical Engineering, North Carolina State University – University of North Carolina at Chapel Hill, Raleigh, Chapel Hill, NC USA; 4 Department of Biostatistics, Medical College of Wisconsin, Milwaukee, WI USA

**Keywords:** Degenerative cervical myelopathy, Virtual reality, Hand dexterity, Post-Surgical rehabilitation, Neuroscience, Outcomes research

## Abstract

Degenerative cervical myelopathy (DCM), the leading cause of non-traumatic spinal cord injury, frequently results in impaired hand dexterity. While surgical decompression is the primary treatment, over 40% of patients report residual hand disability after surgery. There are no therapies to restore hand function after surgery for DCM. In this single-arm clinical trial, post-surgical DCM participants (within 12 months after surgery) underwent a 4-week VR training protocol using the Virtual Keyboard system, which promotes practice of finger individuation. Assessments of hand dexterity were performed at baseline (at week 1), post-training (at week 6) and follow-up (at week 10). The primary outcome measure for hand dexterity assessment was the Jebsen-Taylor Hand Function Test (JTHFT). Twenty-two post-surgical DCM participants were included in the final analysis. Statistically significant improvement in the JTHFT was observed at both post-training (*p* < 0.001, Δ= -15.21s) and follow-up (*p* < 0.001, Δ= -17.84s), with changes exceeding the Minimal Clinically Important Difference (MCID) at both time points. VR hand training also produced significant, sustained and clinically meaningful improvements in quantitative hand dexterity tests and health-related quality of life. The results of this uncontrolled, single-arm study demonstrate the feasibility and efficacy of targeted neurorehabilitation to augment post-surgical neurological recovery in people with DCM.

**Supplementary Information:**

The online version contains supplementary material available at 10.1038/s41598-025-05793-5.

## Introduction

Degenerative cervical myelopathy (DCM), the most prevalent cause of non-traumatic spinal cord injury in adults, affects approximately 2% of the adult population^1,2^. DCM is a progressive disorder characterized by chronic compression of the spinal cord due to osteoligamentous degeneration of the cervical spine^3^. Impaired hand dexterity is seen in over 70% of individuals presenting with DCM^4^. Surgical decompression is the primary treatment for DCM, but recovery is often incomplete. Over 40% of patients report persistent upper limb disability following surgery^5^. Due to its profound effect on quality of life, recovery of upper limb function is one of the top recovery priorities for persons living with DCM^6^.

At present, there are no standardized protocols or targeted rehabilitation strategies to restore hand function for DCM; few studies have been performed to evaluate post-surgical therapy in DCM^7^. Post-surgical rehabilitation treatment for DCM is largely based on experience from other neurological conditions such as traumatic spinal cord injury and stroke. In clinical practice, conventional occupational therapy is often prescribed for residual post-surgical hand dysfunction in DCM. For upper limb rehabilitation, therapy typically includes a combination of interventions such as stretching exercises to improve flexibility and reduce spasticity, grasp-and-release exercises to enhance fine motor control and dexterity and strengthening exercises to rebuild grip strength and endurance^8,9^. Conventional occupational therapy is dependent on the ability of the therapist to maintain engagement and compliance. Repetition intensity and dose of conventional therapy, however, has been shown to be insufficient to achieve plasticity-based motor recovery^10–12^. Virtual reality (VR) interventions hold promise for upper limb rehabilitation^13^, offering several advantages over conventional therapies, including high repetition rates, individualized training intensities, sensory feedback, and motor learning-based approaches^13^. VR training has been tested for chronic neurological conditions such as stroke^14^ and multiple sclerosis^15^ showing superior gains in functional outcomes when compared to traditional occupational therapy. A recent systematic review^16^ showed that only a few studies have used VR training to restore upper limb function in cervical spinal cord injury, highlighting the unexplored potential for VR applications in spinal cord disorders. The Virtual Keyboard (VK) is an innovative VR hand training tool where the impaired hand controls a virtual hand playing a set of keys^17^. The tool promotes finger individuation in an engaging, interactive virtual environment. In a randomized trial^17^, a 6-week training program with an actuated version of the VK system outperformed intensive occupational therapy in chronic stroke patients for hand task performance at one month after training. The VK system offers unprecedented flexibility in adjusting task difficulty to match the user’s capabilities, ensuring an optimal level of challenge for motor learning—an ideal approach for DCM patients.

In this pilot study, we aimed to determine the utility of the VK system to restore hand dexterity in DCM. Specifically, the objective of this study was to evaluate the effectiveness of intensive finger dexterity training post-surgery, using the VK system to promote high-intensity practice of finger individuation in an interactive virtual environment. We hypothesized that training with the VK system would improve hand dexterity in post-surgical DCM patients with residual hand impairment.

## Methods

This study was a single-arm, unblinded, clinical trial to test the therapeutic effect of VR hand dexterity training for post-surgical DCM patients with residual hand impairment. The study was approved by the Institutional Review Board at the Medical College of Wisconsin (Approval Number: PRO00044319) and registered with ClinicalTrials.gov (Identifier: NCT06754072) on December 30, 2024. All methods were performed in accordance with the relevant guidelines and regulations, including the Declaration of Helsinki. We followed CONSORT 2010 reporting guidelines with necessary modifications for a non-randomized trial design. Specifically, a customized version of the CONSORT 2010 Flow Diagram was used to account for the single-arm, non-randomized nature of the study.

### Participants

Post-surgical (within 12 months of surgery) DCM patients were recruited for this study from a tertiary academic medical center. Participants with the following conditions were excluded: pregnancy, epilepsy, history of brain surgery, upper extremity surgery within the last year, significant arm/hand pain limiting movement, or complete paralysis of the hands (upper limb modified Japanese Orthopaedic Association (mJOA) score = 0). We also excluded candidates with other neurologic and muscular diseases, as these conditions could affect both participant attitudes and performance on quantitative tests of hand function. Participants were allowed to participate in conventional outpatient physical therapy as prescribed by their physician as part of standard of care before, during or after enrollment in the study.

### Protocol

**Fig. 1:**
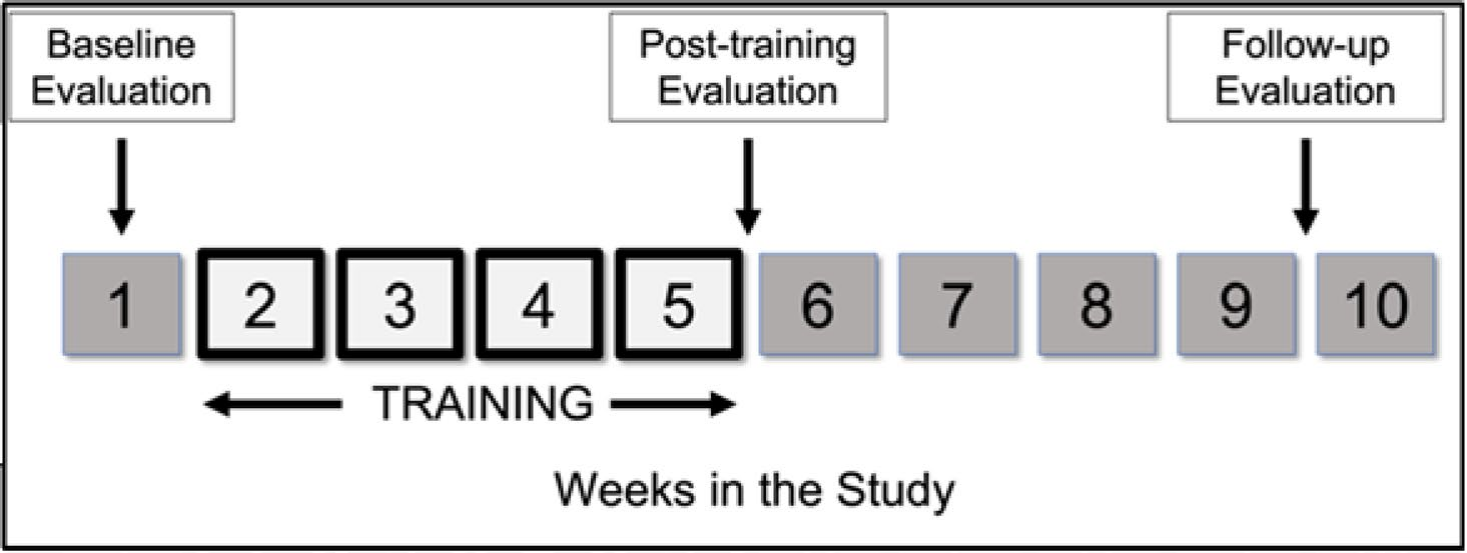
Study design and timeline for each participant. Blocks with dark outlines indicate training weeks. Vertical arrows indicate evaluation sessions.

**Fig. 2:**
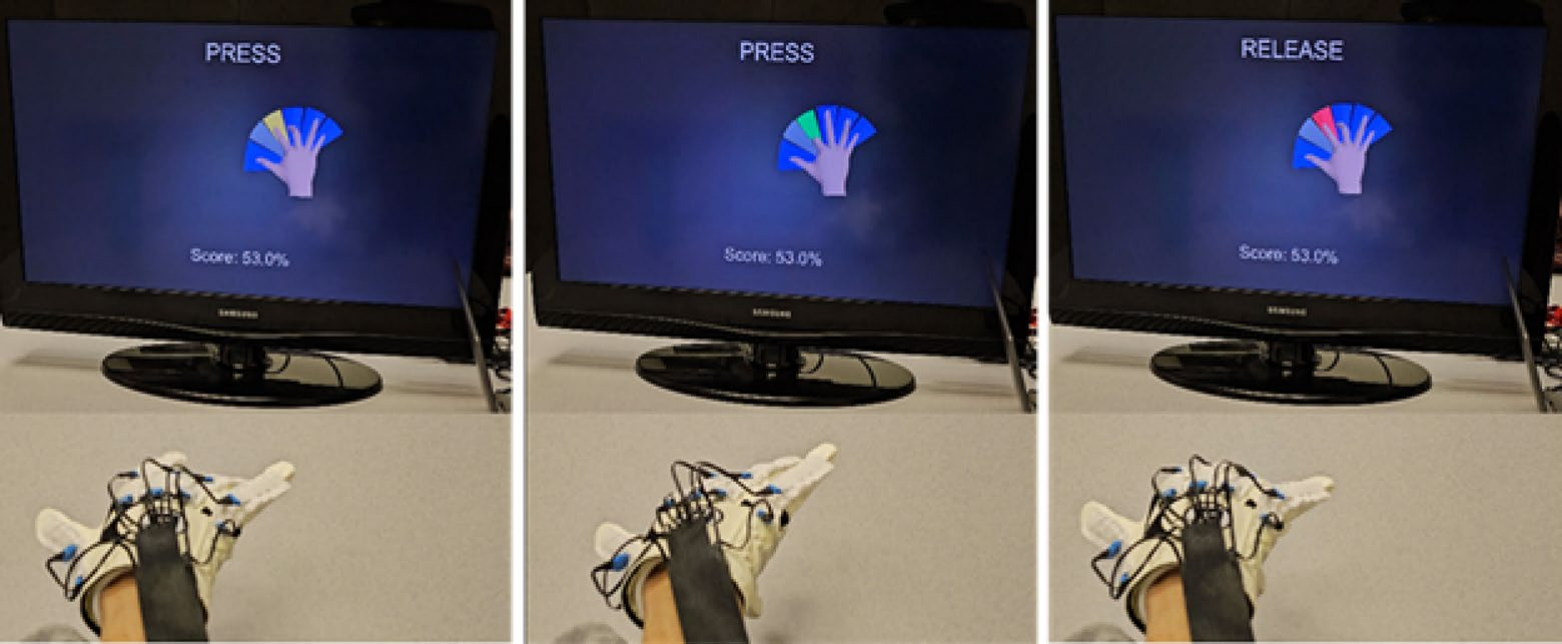
VK system training in progress. The three sections indicate prompts at various stages of training: left- before press, center- during accurate key press, and right- during release. (Note: *Flex* mode has *ready*, *press*, and *release* stages while *Song* mode has only *press* and *release* stages).

Participants were recruited for a four-week training intervention, consisting of three, one-hour sessions per week (Fig. 1). The VK system^17^ combines a custom sensor glove, with a virtual scene consisting of a hand and 5 keys. During each training session, participants wore a sensor glove on their self-reported *more affected* hand, supported by a foam arm rest (Fig. 2). A computer display placed about 50 cm from the participant presented the virtual scene. The sensor glove dynamically measured the metacarpophalangeal (MCP) and proximal interphalangeal (PIP) joints for each finger and the MCP and interphalangeal (IP) joints of the thumb using 2-inch bend sensors (2000–0201, Flexpoint Sensor Systems, Inc) that were located on the dorsal side of the glove. The angle measurements from the sensors were transmitted to the VK system, which updated the corresponding joint angles of a virtual hand in real-time. The virtual digits subsequently played virtual piano keys, with one key associated with each digit. Sufficient finger flexion (beyond a set threshold) resulted in the key being “played” (it moves and emits a tone). For active fingers, the flexion threshold was 25°, while for inactive fingers, the threshold was 30°. The system was controlled by a graphical user interface which allowed the research assistants to alter a number of parameters throughout a session to guide the training^17^. The challenge of the task, for example, was adjusted to the capabilities of the user by changing the speed at which the keys were to be pressed and released, as well as by selecting specific key combinations to be practiced. User performance was calculated using the VK score- which reflects the percentage of correctly executed key presses.

Two different training modes, *Flexion* mode and *Song* mode, were employed in each training session. Each session had 5 blocks – 2 blocks of *Flexion* mode and 3 blocks of *Song* mode, with each block lasting 5 min. In the *Flexion mode*, the participants attempted to play discrete key combinations specified on the computer screen one key at a time. This involved depressing one specified virtual key while refraining from depressing the others, then holding this key in the depressed position for a designated amount of time, and finally releasing them when specified. Visual displays guided the participant (e.g., the key to be played turned yellow; an accurate key press was indicated in green; and a key release or inaccurate key press was shown in red; see Fig. 2). Each key was associated with a unique tone, which played whenever the key was struck. A computer algorithm tracked which combinations were most difficult for the participant and adjusted the presentation of specific combinations according to performance. In the *Song Mode*, participants attempted to play 5-note songs, such as “*Happy Birthday*,” as guided on the computer screen. The user played keys sequentially in the specified manner (Fig. 2 – only *press* and *release* in *song* mode) to create the appropriate pitch and rhythm for the song. Thus, the appropriate key (and only that key) needed to be struck at the appropriate time. A running VK score based on performance was displayed to the participant. The score reflected the percentage of correctly executed presses and releases in the *Flexion* mode, and the percentage of notes played correctly in the *Song* mode. The score was reset at the end of each song; participants were encouraged to attempt to increase their score each time they played a song.

Training difficulty was dynamically adjusted using the *difficulty* parameter. *Difficulty* was the percentage of the available press time during which the participant was required to maintain the correct finger posture^17^. Decreasing *press duration* sped up the game, requiring faster responses. *Press duration* was decreased by half a second each week (from 4 s to 2.5 s), and *difficulty* was adjusted based on the VK score (VK score < 40 → decrease by 10, VK score > 70 → increase by 10). During each week, participants were required to individuate their fingers for an average of 375, 425, 500, and 600 times per session in weeks 1, 2, 3, and 4, respectively.

### Outcome measures

Outcomes were recorded prior to the start of training (baseline), immediately following the end of the 4-weeks of training (post-training) and at 4 weeks after training ended (follow-up) (Fig. 1). The primary outcome measure was the Jebsen-Taylor Hand Function Test (JTHFT)^18^. Secondary quantitative tests of hand dexterity such as the Nine-Hole Peg Test (9HPT)^19^ and Box and Blocks Test (BBT)^20^ were also performed. Sensorimotor hand function was assessed for 3-point pinch strength using a pinch gauge (PG-60, B&L Engineering) and touch sensitivity of the palmar and dorsal hand using von Frey filaments (Aesthesio^®^). Touch sensitivity was scored out of 12, where 12 indicated full sensation. A number of questionnaires and clinical assessments were performed as well: upper limb disability questionnaire (QuickDASH^21^), quality-of-life indicators (EuroQol 5 Dimensions Level Sum Score (LSS) & Visual Analog Scale (VAS)^22^, SF-36v2^23^ Physical and Mental Component Scores (PCS & MCS), myelopathy-specific scores (Myelopathy Disability (MDI)^24^ and modified Japanese Orthopedic Association scale (mJOA)^25^.

To evaluate participants’ perceptions of the training experience, a customized Likert scale feedback form was administered at the conclusion of the training sessions. The form included five statements regarding the training experience, each rated on a scale from 1 (Strongly Disagree) to 5 (Strongly Agree). The statements were: (1) “I am satisfied with the training experience,” (2) “The training met my expectations,” (3) “The training was engaging and helpful,” (4) “I prefer VR-based training over other forms of therapy,” and (5) “The VR-based training addressed my hand symptoms to a greater extent compared to other forms of therapy.”

### Data analyses

Since no similar study had been conducted with DCM participants, a power analysis (G*Power 3.1 software^26^) was performed using data obtained from a pilot study with chronic stroke survivors^17^. In that study, participants who used the actuated VK system exhibited an average decrease of 32.9 ± 50.9 s (mean ± standard deviation) from baseline to the one-month follow-up evaluation session for time to complete the JTHFT^17^. We aimed for a sample size of 21 participants to detect an effect size of 0.64 with 80% power and α = 0.05. To account for potential attrition (15%), we planned to recruit 25 participants for this study.

Data were sourced from records of participants at three time points: baseline, post-training, and follow-up. A set of 14 assessments, including clinical and functional measures, was defined for analysis. Descriptive statistics for the changes (Δ) at post-training and follow-up were calculated to assess the improvement/deterioration in outcome measures compared to baseline. We calculated the Minimum Clinically Important Differences (MCID) using a distribution-based method, where MCID (-11.3 s) was set to 0.5 times the standard deviation (SD) of the baseline values for each outcome measure^27^. To validate clinical relevance of this threshold, we performed a secondary anchor-based MCID analysis using the mJOA score (change of ≥ 1 point^28^), which showed a similar MCID (-11.5 s) provided the optimal discrimination between improved and non-improved participants, thereby validating the clinical relevance of this threshold (see Figure A1 – additional file 1). We calculated the Minimum Clinically Important Differences (MCID) using a distribution-based method. Specifically, we set MCID equal to 0.5 times the standard deviation (SD) of the baseline values for each outcome measure^27^. This approach was adopted due to the lack of established MCIDs in the DCM population.

To evaluate changes in various clinical outcome measures over the three timepoints (baseline, post-training, and follow-up), we employed mixed-effects modeling for repeated measures, specifically a mixed-effects regression analysis. This approach allowed us to account for both fixed (variability over time) and random effects (variability by participant). For each outcome measure, a separate mixed-effects model was fitted with time as a categorical variable. Baseline was set as the reference category to allow direct comparisons between post-training and follow-up against the baseline. The mixed-effects models were estimated using restricted maximum likelihood (REML). Analyses were conducted using Python’s *statsmodels* package and p-values for the post-training and follow-up timepoints were extracted to assess whether significant changes occurred relative to baseline.

Sub-group analyses were performed based on age (< 65 yrs and > 65yrs), time since surgery (0–3 months and 3–12 months), sex (male and female), baseline mJOA severity (mild and moderate-severe), chosen therapy hand (dominant and non-dominant), and start of outpatient PT (started PT before VR study and started PT after VR study/ received no PT). An age threshold of 65 years was used, as patients under 65 have been shown to experience better postoperative functional recovery and quality of life improvements compared to those over 65^29^. We calculated descriptive statistics for changes in JTHFT scores at post-training and follow-up compared to baseline and used Mann-Whitney U tests to assess these changes across subgroups based on normality tests. Statistical significance was set at *p* < 0.05.

## Results

**Fig. 3:**
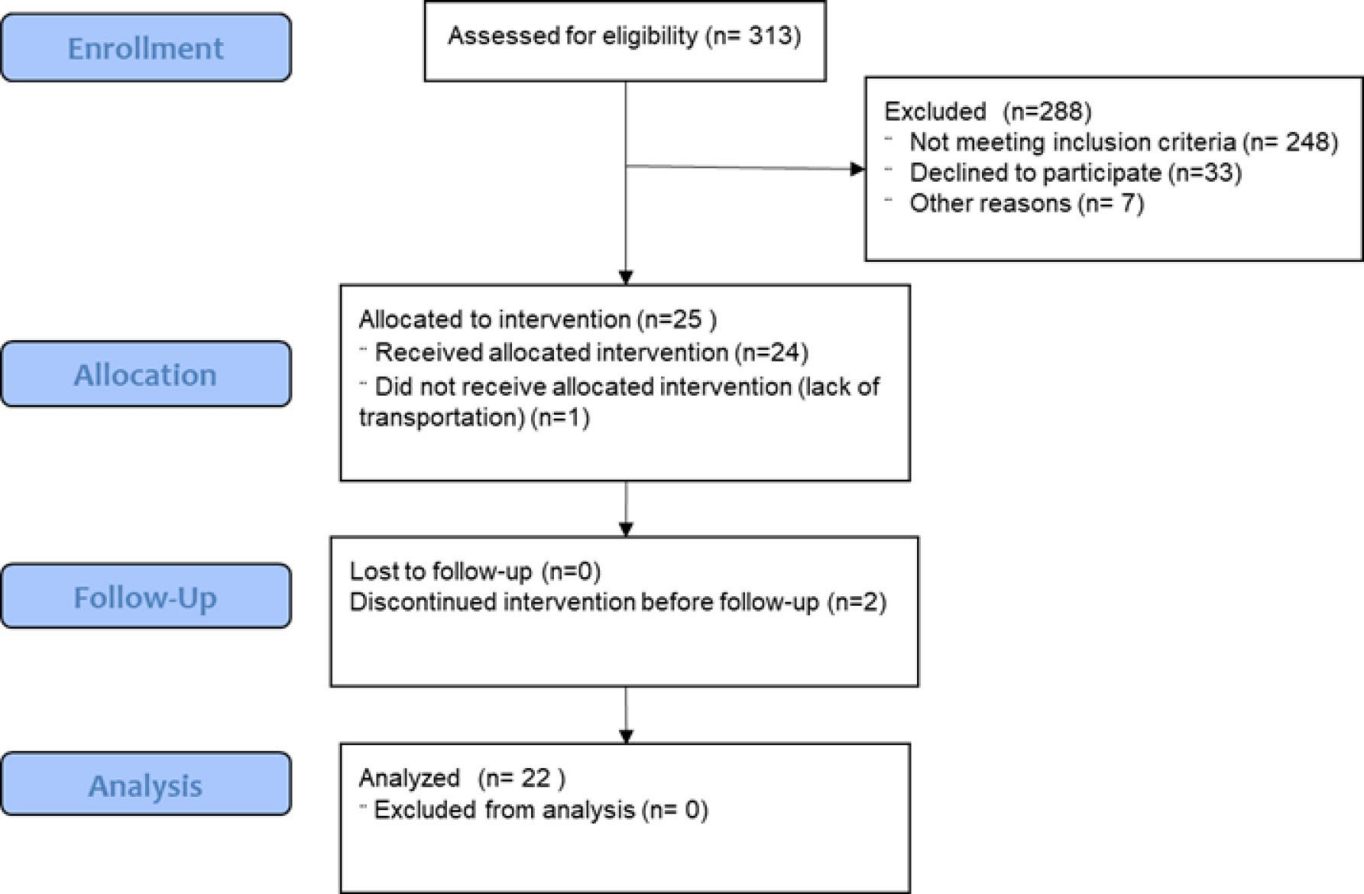
CONSORT 2010 Flow Diagram- modified for non-randomized trial design.

The CONSORT flow diagram^30^, modified for a non-randomized trial, is shown in Fig. 3. Baseline demographics and clinical data are shown in Table 1. A total of 22 participants (*n* = 22/25, 88%), 9 females (40.9%) and 13 males (59.1%), completed the protocol and were included in the final analysis. The mean (SD) age of the participants was 65.3 (11.0) years. The cohort had a mean baseline mJOA of 14.7 (2.2), mJOA upper extremities score of 4.3 (0.8), and mJOA sensation score of 2 (0.5). Surgical treatment for participants in this cohort included anterior and posterior approaches, comprising 17 posterior, 3 anterior, and 2 combined anterior-posterior surgeries, with a mean time since surgery of 4.2 (2.4) months. Surgical levels included C2-T1 (*n* = 1), C3-C4 (*n* = 1), C3-C5 (*n* = 3), C3-C6 (*n* = 2), C3-C7 (*n* = 2), C3-T1 (*n* = 3), C3-T2 (*n* = 2), C4-C5 (*n* = 1), C4-C6 (*n* = 3), C4-C7 (*n* = 2), and C5-C7 (*n* = 2). Twelve participants had received outpatient PT before (*n* = 10) or during (*n* = 2) the VR intervention while the rest of the cohort (*n* = 10) either received PT after (*n* = 6) the intervention or did not receive (*n* = 4) PT. None of the 22 participants received any OT and the PT generally focused on the cervical/ shoulder exercises without any exercises specifically targeting hand dexterity. All participants completed 99.9% of the scheduled training sessions, with only one session missed by a single participant.

**Table 1:** Demographics, severity scores, and time since surgery of study cohort.

Variable	Summary
Total	22
Sex	
Females	9 (40.9%)
Males	13 (59.1%)
Age	
< 65 years	9 (40.9%)
> 65 years	13 (59.1%)
Baseline mJOA	
Mild (15–17)	13 (59.1%)
Severe-Moderate (< 14)	9 (40.9%)
Time since surgery	
0–3 months	8 (36.36%)
3–12 months	14 (63.64%)
Surgery type	
Posterior	
PCDF	7 (31.82%)
Laminectomy	4 (18.18%)
Laminoplasty	6 (27.27%)
Anterior	
ACDF	3 (13.64%)
Anterior- Posterior	
ACDF + PCDF	2 (18.18%)
No. of involved levels	
2	2 (9.09%)
3	8 (36.36%)
4	4 (18.18%)
>4	8 (36.36%)
Therapy hand	
Dominant hand	16 (72.73%)
Non-dominant hand	6 (27.28%)
Outpatient PT	
Started before/during VR	12 (54.55%)
Started after VR/no PT	10 (45.45%)

(Note: PCDF- posterior cervical decompression and fusion, ACDF- anterior cervical discectomy and fusion).

### Post-training

**Fig. 4:**
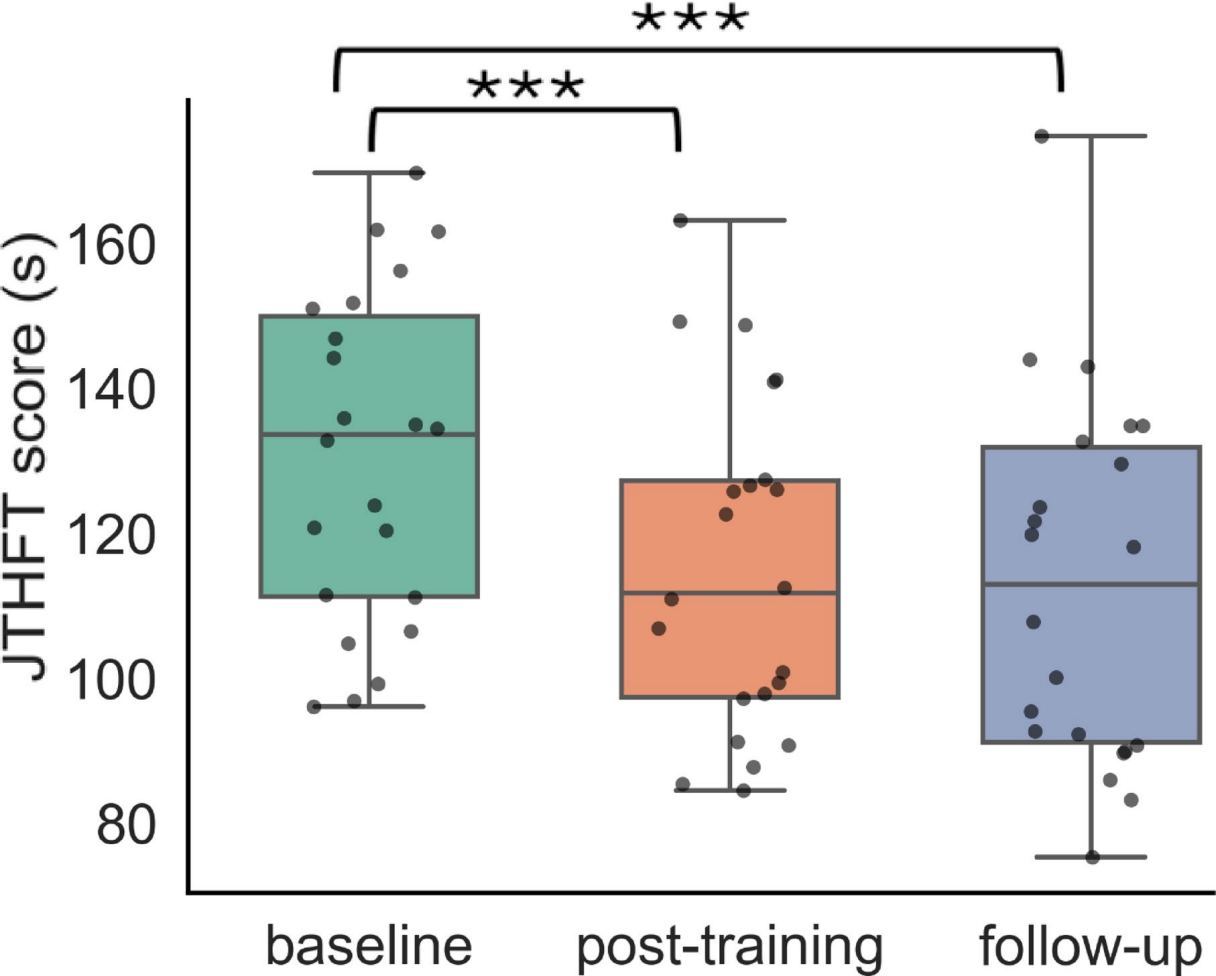
Box plots of our primary outcome measure– JTHFT score at baseline, post-training, and follow-up. (Note: ***significant at α = 0.001).

**Fig. 5:**
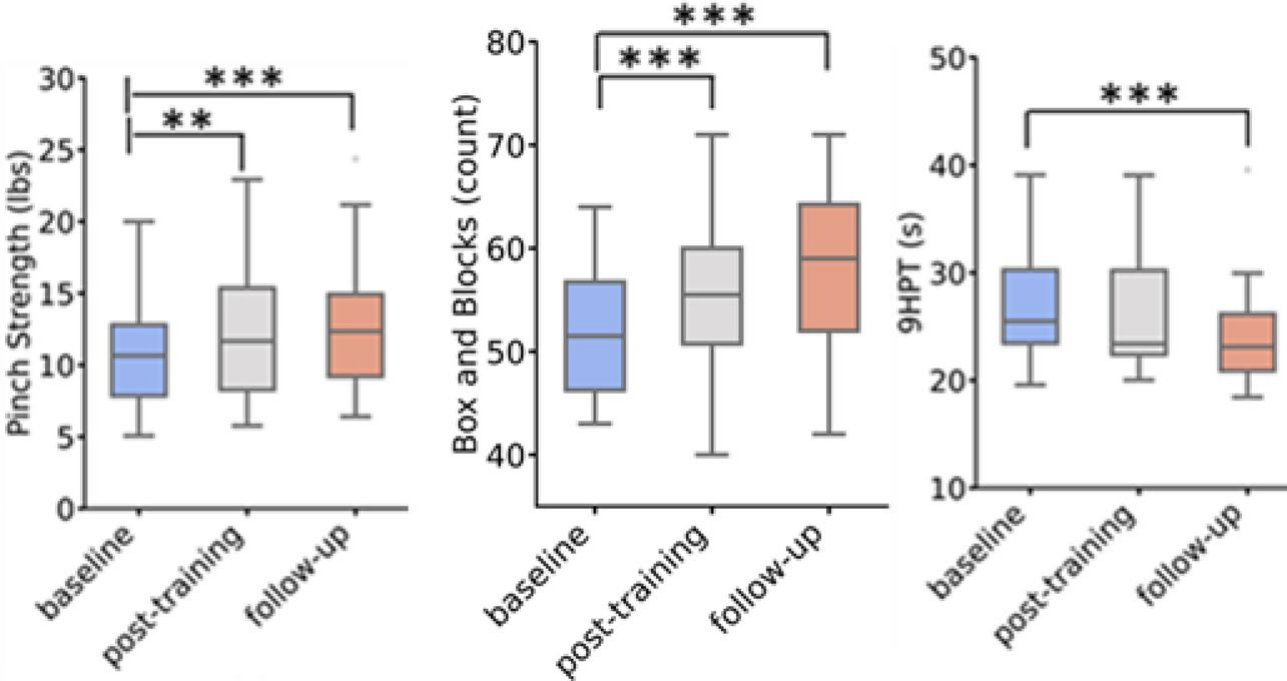
Box plots of clinical measures at baseline, post-training, and follow-up. (Note: *significant at α = 0.05, **significant at α = 0.01, ***significant at α = 0.001).

JTHFT score, the primary outcome measure, showed significant improvement at post-training (*p* < 0.001, Δ= -15.26 s, t= -5.2) and exceeded the MCID threshold (Fig. 4). Several secondary outcome measures demonstrated significant differences at post-training as well (Fig. 5; Table 2, and Fig A2 (see Additional File 1)): pinch strength (*p* < 0.001, Δ = 1.49 lbs., t = 3.4), BBT (*p* < 0.001, Δ = 4.50, t = 4.4), QuickDASH (*p* = 0.008, Δ= -5.79, t= -2.7), MDI (*p* = 0.035, Δ= -1.18, t= -2.8), and mJOA sensation (*p* = 0.005, Δ = 0.27, t = 2.1). The SF36v2 MCS (*p* = 0.069, Δ = 1.77, t = 1.6) showed a trend toward significance. BBT and mJOA sensation scores exceeded the MCID (Table 2).

**Table 2:** Changes in outcome measures at post-training and follow-up with MCID assessment and p-values from the mixed-effects model.

Outcome Measure	Δ post-training mean [95% CI]		Δ follow-up mean [95% CI]	MCID	post-training t- statistic	post- training *p*-value	follow-up t-statistic	follow-up *p*-value
JTHFT (s)	**-15.26 [-21.73**,** -8.79]**		**-17.84 [-23.89**,** -11.80]**	-11.21	-5.2	***<0.001	-6.1	***<0.001
Pinch strength (lbs.)	1.49 [0.70, 2.29]		**2.19 [1.25**,** 3.14]**	1.96	3.4	***<0.001	5.0	***<0.001
BBT	**4.50 [2.40**,** 6.60]**		**5.86 [3.79**,** 7.94]**	3.07	4.4	***<0.001	5.7	***<0.001
9HPT (s)	-0.82 [-2.00, 0.36]		**-2.72 [-3.65**,** -1.80]**	-2.44	-1.4	0.150	-4.8	***<0.001
Touch sensitivity	0.05 [-0.34, 0.43]		0.41 [-0.19, 1.01]	0.58	0.2	0.870	1.5	0.142
mJOA upper	0.09 [-0.08, 0.27]		0.00 [-0.22, 0.22]	0.37	0.9	0.392	0.0	1.000
mJOA sensation	**0.27 [-0.04**,** 0.59]**		**0.36 [0.16**,** 0.57]**	0.26	2.1	*0.035	2.8	**0.005
mJOA total	0.46 [-0.17, 1.08]		0.82 [0.23, 1.40]	1.08	1.5	0.146	2.6	**0.009
MDI	-1.18 [-1.99, -0.37]		-0.73 [-1.72, 0.27]	1.89	-2.8	**0.005	-1.7	0.081
QuickDASH	-5.79 [-10.43, -1.14]		-7.03 [-11.11, -2.94]	-7.36	-2.7	**0.008	-3.2	**0.001
EuroQol5D LSS	-0.55 [-1.62, 0.53]		-1.27 [-2.06, -0.48]	-1.95	-1.1	0.265	-2.6	**0.009
EuroQol5D VAS	3.86 [0.04, 7.69]		6.46 [1.43, 11.48]	10.61	1.6	0.108	2.7	**0.007
SF-36v2 PCS	1.77 [-0.40, 3.93]		2.58 [0.15, 5.00]	3.19	1.6	0.110	2.3	*0.020
SF-36v2 MCS	3.45 [-0.58, 7.48]		5.98 [2.32, 9.64]	7.52	1.8	0.069	3.2	**0.002

(Note: **bold** indicates Δ > MCID/ significant p-value; *significant at α = 0.05, **significant at α = 0.01, ***significant at α = 0.001).

### Follow-up

Significant improvements at follow-up were noted for the JTHFT (*p* < 0.001, Δ= -17.89s, t= -6.1) at follow-up. Significant improvement in secondary outcome measures, such as pinch strength (*p* < 0.001, Δ = 2.19 lbs., t = 5.0), BBT (*p* < 0.001, Δ = 5.86, t = 5.7), 9HPT (*p* < 0.001, Δ= -2.72s, t= -4.8), QuickDASH (*p* = 0.001, Δ= -7.03, t=-3.2), mJOA total (*p* = 0.009, Δ = 0.82, t = 2.6), and sensation (*p* = 0.005, Δ = 0.36, t = 2.8). EuroQol5D metrics were significantly improved for LSS (*p* = 0.009, Δ= -1.27, t= -2.6) and VAS (*p* = 0.007, Δ = 6.46, t = 2.7). The SF36v2 components demonstrated improvements in mental health (SF36v2 MCS, *p* = 0.002, Δ = 5.98, t = 3.2) and physical health (SF36v2 PCS, *p* = 0.020, Δ = 2.58, t = 2.3). The improvements in JTHFT, BBT, mJOA sensation, 9HPT, and pinch strength exceeded the MCID at the follow-up assessment (Table 2).

### Subgroup analyses

**Fig. 6:**
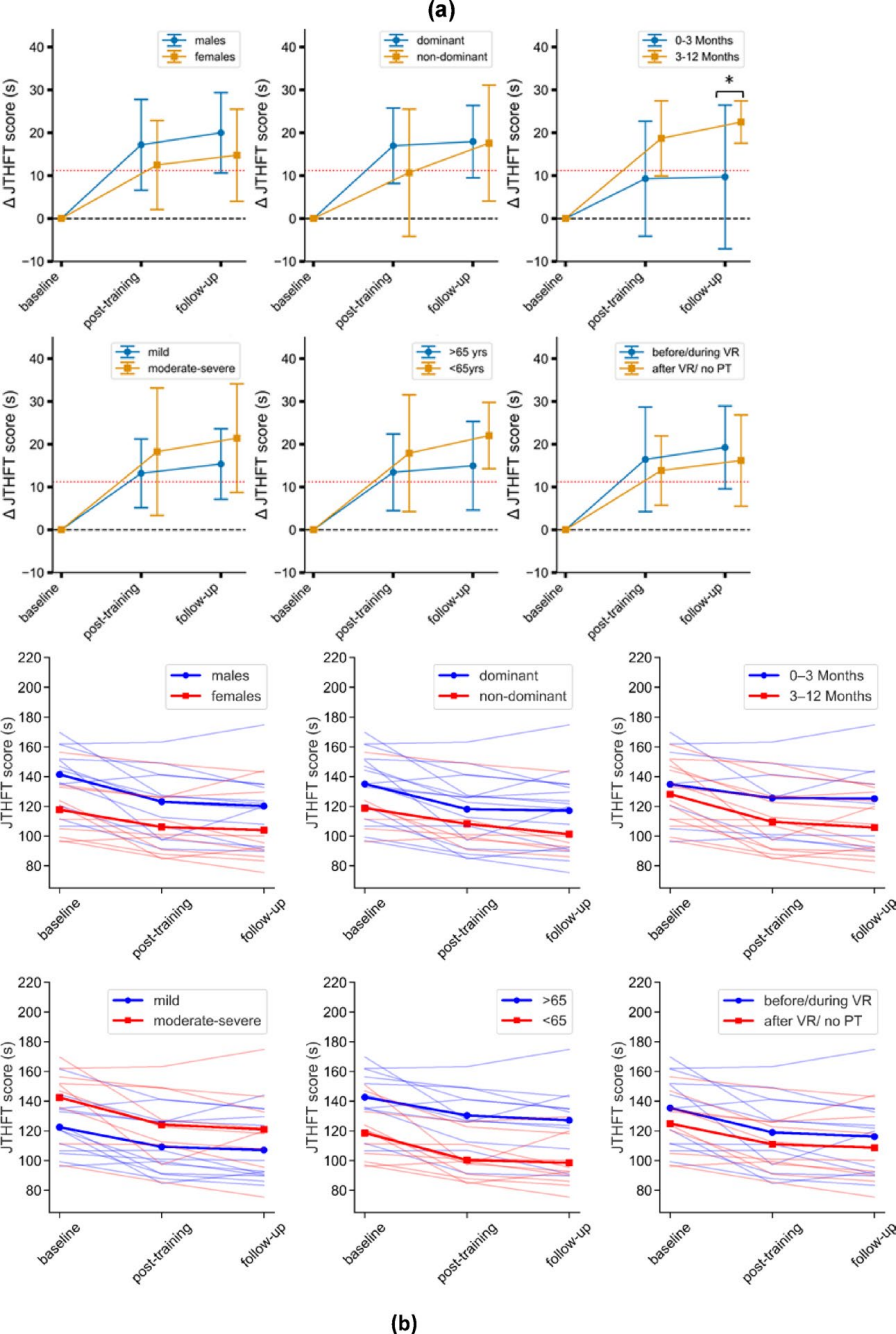
(**a**) Assessing the impact of sex, choice of therapy hand, time since surgery, DCM severity, age, and timing of outpatient PT on improvements in JTHFT scores across time points. (Note: MCID is depicted as a red dotted line. Mean [95% CIs] JTHFT changes are shown as positive here. *significant at α = 0.05). (**b**) Individual -level trajectories (spaghetti plots) of JTHFT scores over time, grouped by sex, choice of therapy hand, time since surgery, DCM severity, age, and timing of outpatient PT. Transparent lines represent individual trajectories, while bold lines denote the group average trajectories for each sub-group.

Subgroup analyses (see Table A1- Additional File 1) to detect the impact of sex, choice of therapy hand, time since surgery, mJOA severity, and age on improvement in the primary outcome (JTHFT) are shown in Fig. 6a and b. Improvement in JTHFT scores was significantly greater at follow-up for patients (*n* = 14) who were 3–12 months post-surgery at baseline as compared to patients (*n* = 8) who were less than 3 months post-surgery at baseline (*p* = 0.026, U = 89). At the post-training time point, improvement in JTHFT between these subgroups approached statistical significance (*p* = 0.070, U = 83). No significant differences were observed for the other subgroups: age (*p* = 0.525, 0.306), sex (*p* = 0.402, 0.275), choice of therapy hand (*p* = 0.449, 0.712), mJOA severity (*p* = 0.548, 0.333), or outpatient PT (*p* = 0.869, 0. 767) for post-training and follow-up, respectively.

### Feedback

The feedback provided by the participants on the VR training experience was positive. The mean (SD) scores were 4.73/5 (0.5) for overall satisfaction, 4.5/5 (0.7) for meeting their expectations, and 4.55/5 (0.6) for engagement and helpfulness. Participants preferred VR-based training over other therapies (3.91/5 (0.8)) and rated its effectiveness in addressing hand symptoms as 3.82/5 (0.8). Qualitative feedback included participants highlighting areas for improvement, such as enhancing system responsiveness, reducing delays, and resolving issues like sudden screen flashes and incorrect notes in some songs. Some participants noted differences from a normal piano, such as the pressing action requiring “*bending rather than direct pressing”* and expressed mixed opinions about the use of gloves and sensors. Despite these challenges, one participant reported positive effects, including “*improved muscle activation in the thumb”.*

## Discussion

This study is the first prospective clinical trial to demonstrate the efficacy of post-surgical rehabilitation for hand function in DCM. Twenty-two post-surgical DCM participants (within 12 months after surgery) underwent a 4-week (12 sessions) VR training paradigm for their impaired hand. We demonstrated statistically significant improvements in objective measures of hand function (JTHFT, BBT, pinch strength) as well as quality of life (EuroQoL5D, SF36v2). The improvements in hand function (JTHFT and BBT) exceeded the MCID scores at 4 weeks follow-up indicating that the training contributed to meaningful, sustained recovery of hand function.

Our findings demonstrated that participants engaging in the VK training exhibited significant improvements in hand function, as evidenced by the JTHFT, BBT, and pinch strength scores, both immediately post-training and at the 4-week follow-up. The effects of the 4-week training paradigm were sustained for at least one month after the end of the training (the time of the follow-up evaluation), thus suggesting a durable treatment response. Other secondary outcome measures, including 9HPT, QuickDASH, MJOA sensation, and various quality-of-life indicators (EuroQoL5D LSS & VAS, SF36v2- MCS & PCS), also showed improvement at follow-up. These results suggest that enhancing hand dexterity can lead to broader functional gains and improved hand use during activities of daily living for DCM patients. These results are consistent with previous studies^31–34^ utilizing VR interventions for upper limb rehabilitation, which reported improvement in motor function. Future studies are necessary to determine how hand dexterity training translates into increased hand use during daily activities and if this effect is sustained at longer term follow-up.

VR training led to significant greater improvement in hand dexterity, as measured by the JTHFT, for patients 3–12 months after surgery compared to those less than 3 months after surgery. The improvement in JTHFT did not meet MCID in the subgroup undergoing VR training less than 3 months after surgery but did achieve MCID in patients who were 3–12 months after surgery. In a prior study, Rahman et al.^35^ showed that initiating conventional occupational therapy within six weeks of surgery led to greater improvements in quality of life (SF-36v2 PCS) at one year compared to delaying the start of therapy. This discrepancy may stem from the different outcome measures used, where functional assessments like the JTHFT detect subtle changes in motor performance compared to quality-of-life scores such as the SF-36v2 PCS. Variability in recovery trajectories, adherence to therapy, and differences in rehabilitation protocols can also influence these outcomes. Previous studies emphasize that recovery of hand function following surgery can be gradual, with full recovery not always achieved immediately^4,5^. Machino et al.^5^ found that while many patients experience improvement after surgery, residual symptoms, including hand function deficits, often persist and may take several months to improve. Chiles et al.^4^ reported that deficits, particularly in fine motor skills, improve gradually, with significant progress typically observed within the first 6 months after surgery. Our results indicate that VR can lead to improvements in hand dexterity beyond the first 3 months after surgery, a time point at which spontaneous neurological recovery after surgery is slow^36^.

The VK system was originally tested on stroke survivors^17^. We adapted this device for use with post-surgical DCM participants for this clinical trial. Specifically, we removed the actuation, which made the tool much easier to doff and don. The resulting VK system encourages a high volume of repetitive movements while requiring continuous finger individuation, thereby leading to intensive practice of fine motor tasks. This distinctive feature allows for constant refinement of specific motor skills, making it particularly advantageous for individuals with minimal motor weakness but impaired dexterity, such as DCM patients. The VK system provides an engaging environment that integrates sensory information during therapy and has the ability to motivate users through gamification with automated recording of motor performance and compliance with therapy. Compliance in this study was good with 88% (*n* = 22/25) of enrolled participants completing the full protocol.

The observed improvements in hand function following VR-based training likely stem from a combination of task specificity, high repetition intensity, and gamification. The tasks performed during VR training were unrelated to the JTHFT, yet we noted significant improvements in the JTHFT after VR training. This highlights a generalized improvement in motor control that could be adapted for everyday tasks that are tested on the JTHFT. Participants were required to individuate their fingers an average of 9,200–9,500 times during the therapy. This repetition intensity may have contributed to functional gains through mechanisms of use-dependent plasticity^37,38^. The gamified interface likely enhanced engagement and adherence, reinforcing consistent motor practice. A previous study using the AVK system^17^ in stroke survivors reported that improvements in fine motor function were greater than those achieved through intensive, targeted occupational therapy. DCM is characterized by loss of fine motor hand function and currently there is no standardized therapy, yet occupational therapy is often prescribed for DCM in the post-surgical setting. A prior study showed that post-surgical occupational therapy was associated with significant improvement in mJOA scores at 12 months after surgery^35^, however, that study did not quantify hand impairment. A future randomized controlled trial with a standard occupational therapy control group, a common outcome measure, and longer-term follow-up (e.g., 6–12 months) is needed to assess the durability and clinical impact of the intervention.

The results of the Likert scale feedback highlight the overall positive reception of the VR training among participants, particularly in terms of satisfaction and perceived engagement. The high mean scores suggest that the VR training not only met but exceeded participants’ expectations, aligning well with current trends favoring innovative therapeutic approaches in rehabilitation^13^. However, the preference for VR training over traditional therapies received a slightly lower score, potentially due to the limited gamification of the present device The lack of sufficient gamification could result in reduced participant motivation and engagement, which is crucial for rehabilitation success^39,40^. Participant feedback emphasized the importance of refining VR interventions and tailoring training paradigms to fit patient need. Future studies could focus on exploring specific aspects of VR training that contribute to its effectiveness, ensuring that it serves as a robust complement to conventional rehabilitation strategies.

This study has several limitations. This study was designed as a single arm clinical trial and a comparative trial between VR and conventional occupational therapy for DCM is yet to be performed. Blinding of the assessors may further reduce bias in the outcome assessments. Longer follow-up duration is necessary to determine durability of treatment response. Currently, the VK system is laboratory-based and uses an instrumented glove fitted to the participant’s hand size. Future iterations of the device would include wireless hand tracking and home-based rehabilitation to improve accessibility and enhance clinical adoption. Another aspect of improvement could involve enhancing gamification features to boost engagement and extracting predictive markers from in-game performance to predict treatment response and guide personalized therapy. Although the study was adequately powered for the primary outcome, the smaller number of patients for subgroup analysis may have impacted our ability to detect statistical differences. Not all patients benefited from the training, and more research is necessary to identify which participants would benefit most from VR training after surgery for DCM.

## Conclusion

Post-surgical DCM patients showed significant, sustained, and clinically meaningful improvements in hand dexterity and quality of life after participating in a 4-week virtual reality hand training paradigm. The results demonstrate the efficacy of a targeted neurorehabilitative intervention to augment neurological recovery after surgery for DCM. VR hand dexterity training is a novel approach to target residual hand disability after surgery for DCM.

## Electronic supplementary material

Below is the link to the electronic supplementary material.

Supplementary Material 1

## Data Availability

Summary results and data supporting the findings of this study are available from the corresponding author upon reasonable request, subject to ethical and institutional approvals.
