# Supplementary material for: Virtual reality-based training to augment recovery of hand dexterity after surgery for degenerative cervical myelopathy
Source: Sci Rep. 2025 Jul 2;15:22785. doi: 10.1038/s41598-025-05793-5 (PMC12217608; doi:10.1038/s41598-025-05793-5)
Supplement: Supplementary file 1 — Supplementary Material 1 [file 41598_2025_5793_MOESM1_ESM.pdf]

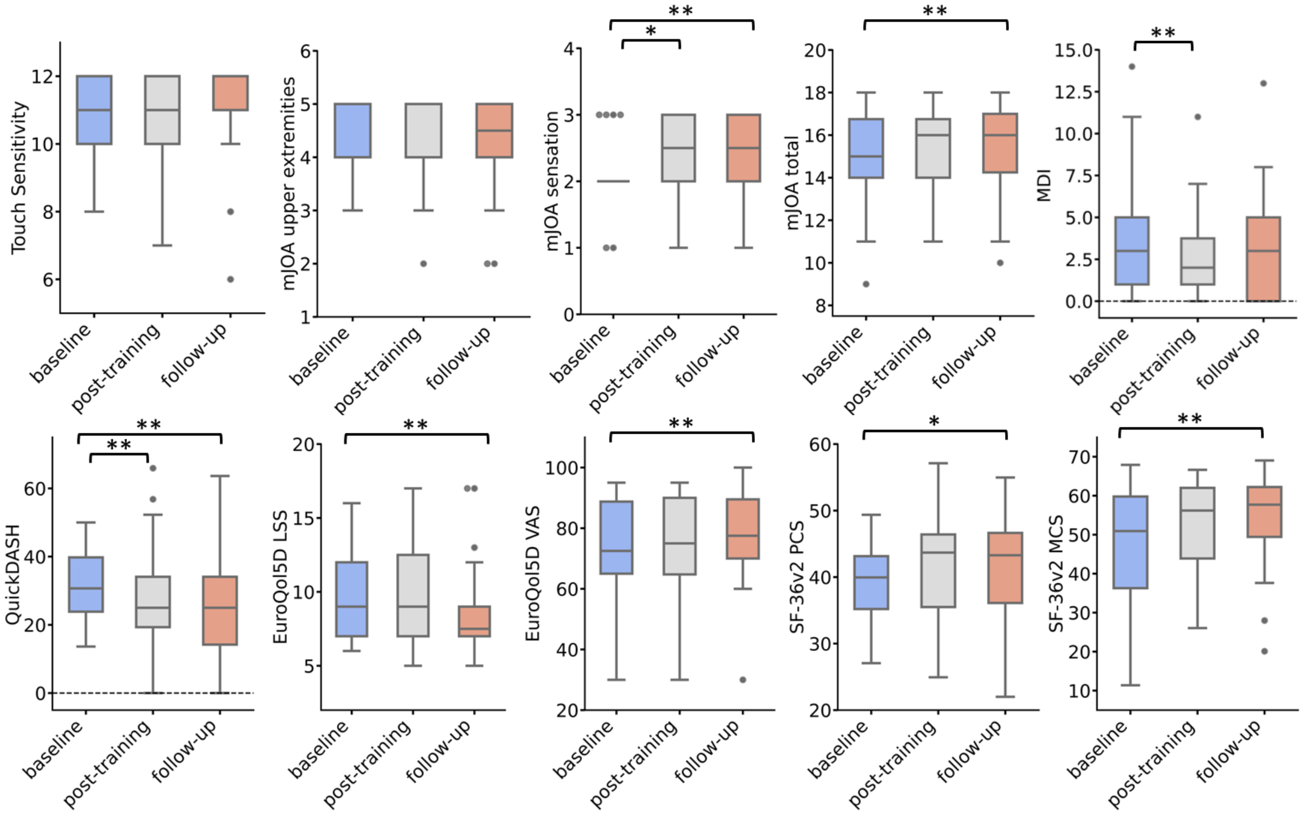

Figure A1: Box plots of clinical measures at baseline, post-training, and follow-up. (Note: *significant at α=0.05, **significant at α=0.01, ***significant at α=0.001)

Table A1: Mann-Whitney U-test results for subgroup analyses (Note: *significant at p=0.05)

Subgroup	Post-training		Follow-up	
	U-statistic	p-value	U-statistic	p-value
**Age**<br><65 (n=9)<br>>65 (n=13)	46	0.525	40.5	0.306
**Time since surgery**<br>0-3 months (n=8)<br>3-12 months (n=14)	83	0.070	89	***0.026**
**Sex**<br>Male (n=13)<br>Female (n=9)	69	0.402	72.5	0.275
**Choice of therapy hand**<br>Dominant (n=16)<br>Non-dominant (n=6)	59	0.449	53.5	0.712
**mJOA severity**<br>Mild (n=13)<br>Moderate-severe (n=9)	68	0.548	73.5	0.333
**Outpatient PT**<br>Before/ during VR (n=12)<br>After VR/ no PT (n=10)	57	0.869	65	0.767
